# Molecular Mechanisms of Phosphorus Metabolism and Transport during Leaf Senescence

**DOI:** 10.3390/plants4040773

**Published:** 2015-12-16

**Authors:** Kyla A. Stigter, William C. Plaxton

**Affiliations:** 1 Department of Biology, Queen’s University, Kingston, Ontario K7L 3N6, Canada; 2 Department of Biomedical and Molecular Sciences, Queen’s University, Kingston, Ontario K7L 3N6, Canada; E-Mail: plaxton@queensu.ca

**Keywords:** leaf senescence, nutrient remobilization, phosphorus-use efficiency, phosphorus metabolism, phosphate transport, phosphodiesterase, purple acid phosphatase, (ribo)nuclease

## Abstract

Leaf senescence, being the final developmental stage of the leaf, signifies the transition from a mature, photosynthetically active organ to the attenuation of said function and eventual death of the leaf. During senescence, essential nutrients sequestered in the leaf, such as phosphorus (P), are mobilized and transported to sink tissues, particularly expanding leaves and developing seeds. Phosphorus recycling is crucial, as it helps to ensure that previously acquired P is not lost to the environment, particularly under the naturally occurring condition where most unfertilized soils contain low levels of soluble orthophosphate (Pi), the only form of P that roots can directly assimilate from the soil. Piecing together the molecular mechanisms that underpin the highly variable efficiencies of P remobilization from senescing leaves by different plant species may be critical for devising effective strategies for improving overall crop P-use efficiency. Maximizing Pi remobilization from senescing leaves using selective breeding and/or biotechnological strategies will help to generate P-efficient crops that would minimize the use of unsustainable and polluting Pi-containing fertilizers in agriculture. This review focuses on the molecular mechanisms whereby P is remobilized from senescing leaves and transported to sink tissues, which encompasses the action of hormones, transcription factors, Pi-scavenging enzymes, and Pi transporters.

## 1. Introduction

Phosphorus (P) is a crucial plant macronutrient, as it is a structural constituent of essential biomolecules involved in both energy metabolism, such as ATP and PPi, and in the formation of key macromolecules such as nucleic acids and phospholipids. Thus, P is integral to nearly all major metabolic processes in plants, including photosynthesis and respiration. Soluble orthophosphate (PO_4_^3−^; Pi), which is the only form of plant-available P that roots can directly assimilate from the soil, is often highly limiting in the natural environment, prompting the widespread use of Pi-containing fertilizers in agriculture [1]. Although fertilizers are effective in bolstering crop yields, only 15%–30% of applied P is typically absorbed by crops in the year of its application [2]. The resulting Pi-runoff from fertilized fields leads to nutrient overloading of aquatic ecosystems, triggering toxic algal blooms and eutrophication of the affected waterways. Furthermore, the Pi contained within these fertilizers is manufactured from non-renewable rock-phosphate reserves, which have been projected to be depleted within the next 80 years [3]. It becomes clear that an alternative and sustainable approach is required to reduce agriculture’s dependence on Pi fertilizers. This may be achieved through the manipulation of the crops themselves by either improving the: (i) ability of their roots to assimilate Pi from the soil, relating to P-acquisition efficiency, and/or (ii) proficiency of Pi recycling within the plant itself, known as P-use efficiency (PUE) [4]. One promising area for crop PUE improvement is to enhance the efficiency of Pi remobilization from senescing leaves to younger sink tissues, particularly developing seeds and immature leaves.

### Nutrient Remobilization during Leaf Senescence

During leaf development, a leaf blade emerges and expands to convert solar energy into chemical energy usable by the plant via the process of photosynthesis. However, as the plant matures, the aging leaf experiences greater respiratory costs as well as diminishing light conditions due to self-shading, ultimately resulting in a reduced output of photosynthate. Although the aged leaf is no longer a photosynthetic asset for the plant, its value lies in the pools of nutrients stored within it [4]. As the aging leaf enters its final stage of development, senescence, it redistributes essential nutrients to sink tissues before tissue death occurs. The transition from an uptake-dominated nutrient supply via the roots to salvaging key nutrients from senescing tissues could be vital to the overall fitness of the plant, particularly in environments where it is energetically costly to acquire nutrients from the soil. Therefore, the efficiency of nutrient remobilization from senescing leaves to developing tissues is important so that precious nutrients, such as Pi, are not lost to the environment upon abscission of the fully-senesced leaf. Pi salvage from older leaves has the obvious adaptive value that it reduces the need to take up Pi from the soil that may be poorly available [4]. The initial target of senescence-mediated catabolism is the chloroplast; it is from this organelle that much of the Pi and other nutrients salvaged from a senescing leaf originate [4]. Small senescence-associated vacuoles with intense proteolytic activity accumulate in leaves of several plant species, and although senescence-associated vacuoles appear to play a key role in nitrogen (N) remobilization [4], future research needs to establish their role in Pi remobilization. Indeed, studies on Pi remobilization from senescing leaves are surprisingly scarce relative to studies of N remobilization, although a recent review concerning Pi remobilization has shed light on this understudied, yet critical, topic [5].

With the availability of its entire genome sequence and a host of related genomic tools, *Arabidopsis thaliana* has become a valuable model plant species for studies of nutrient mobilization during senescence. During *Arabidopsis* leaf senescence, the total amount of N decreases by 85% as cellular proteins are catabolized and the resulting amino acids are exported to other tissues [6]. Likewise, total P levels drop by about 75%, while nutrients such as copper, iron, chromium and potassium decrease by greater than 40% during senescence [6,7]. However, not all plants are created equal in their abilities to recycle nutrients (Table 1). Extremophile species growing in severely Pi-impoverished soils, such as *Banksia serrata* and *Hakea prostrata* (harsh hakea) typically recycle 85%–95% of total P from their senescing leaves, whereas species such as soybean (*Glycine max*) and *Acacia truncata* remobilize less than 50% [8,9,10,11]. We hypothesize that species that are highly efficient at remobilizing Pi from their senescing leaves have evolved many adaptive advantages that allow them to effectively acclimate to the Pi-impoverished soils that they typically inhabit. Such adaptations likely include more effective hydrolytic enzymes, such as nucleases and phosphatases, which play a key role in liberating Pi from macromolecules and/or low molecular weight Pi-monoesters and anhydrides during senescence. Interestingly, while there is a wealth of data surrounding P resorption efficiency for species living in extreme environments, such as the highly Pi-deficient soils of Western Australia and the severe sub-arctic climate, little information exists regarding P resorption efficiencies of common crop species cultivated under typical agricultural conditions. It would be of great interest to perform a comprehensive survey that compares P resorption efficiencies of senescing leaves for various crop species, particularly due to the possibility that traits enabling PUE may have been compromised in modern crop varieties as a byproduct of selecting for maximal yields with maximal fertilizer inputs. Although fertilizer use promotes crop growth and high yields, a global trend has emerged indicating that the greater the green-leaf nutrient concentration, the less efficient the nutrient resorption from senescing leaves will be [12,13]; P resorption efficiency is reduced two-fold with only moderate increases in green-leaf P concentration [13]. As such, the use of fertilizers in agricultural practices may boost efficient crop growth, but could consequently inhibit efficient Pi recycling and thus the overall PUE.

The aim of this article is to provide an overview of our current understanding of the molecular mechanisms that underpin the process of liberating Pi from P-containing molecules, and its remobilization from senescing leaves. This process is compared to the adaptive responses that occur during nutritional Pi-starvation so as to highlight similarities and differences in the regulatory control and molecular mechanisms underlying Pi metabolism during the two conditions. An integrated understanding of Pi remobilization during senescence will facilitate development of effective biotechnological strategies to improve crop PUE, thereby reducing society’s dependency upon polluting and unsustainable Pi-containing fertilizers.

**Table 1:** A comparison of phosphorus (P) resorption efficiencies across a variety of plant species, where P resorption efficiency is defined as the amount of total P resorbed during senescence (expressed as a percentage of the total amount of P present in a fully-expanded, leaf relative to a fully senesced leaf) [14].

Species (Common Name)	Phosphorus Resorption Efficiency (%)	Source
*Acacia truncata* (angle-leafed wattle)	41	[9]
*Acacia xanthine* (white-stemmed wattle)	36	[9]
*Arabidopsis thaliana* (thale cress)	75	[6,7]
*Artabotrys hongkongensis* (talon wild vine)	41	[15]
*Banksia attenuate* (slender banksia)	69	[9]
*Banksia chamaephyton* (fishbone banksia)	82	[16]
*Banksia serrata* (saw banksia)	95	[9]
*Calophyllum polyanthum* (sirpoon tree)	53	[15]
*Cladium jamaicense* (Jamaica swamp grass)	78	[17]
*Empertrum hermaphroditum* (mountain crowberry)	70	[18]
*Eriophorum vaginatum* (tussock cottongrass)	90	[18]
*Glyceria maxima* (reed mannagrass)	22	[19]
*Glycine max* (soybean)	50	[8]
*Hakea prostrata* (harsh hakea)	85	[10,11]
*Michelia floribunda*	80	[15]
*Phragmites australis* (common reed)	50	[19]
*Vaccinium uliginosum* (bog blueberry)	40	[18]

## 2. Transcriptome Changes Promote Phosphorus Remobilization during Leaf Senescence

Several studies have investigated gene expression changes during leaf senescence by analyzing genome-wide transcriptomes of leaves undergoing this final phase of development. These studies have provided a wealth of knowledge through large-scale comparisons of genes and gene families that are up-regulated or down-regulated during the senescence syndrome. In addition, these studies provide insight into how senescence is both initiated and modulated, with potential key genes being identified for further study to advance our understanding of the senescence program. Genome-wide comparisons among various species including *Arabidopsis* [20,21,22,23,24], wheat (*Triticum aestivum*) [25], rice (*Oryza sativa*) [26], maize (*Zea mays*) [27], and cotton (*Gossypium hirsutum*) [28] exhibit an overlap in senescence-related loci, which could indicate relative conservation in the leaf senescence program. Although advancements have been made in identifying regulators of senescence, such as transcription factors and hormones, the downstream targets for many of these regulatory elements have yet to be elucidated. Here, the transcriptomic response of regulatory elements known to influence downstream targets that function in Pi remobilization during leaf senescence are explored.

### 2.1. Differential Gene Expression over the Course of Senescence

To make the transition from a photosynthetically active organ to senescing tissue requires a change in the genetic program being actively expressed; this involves the integration of both hormones and transcription factors to signal downstream protein targets that provide the biochemical machinery to remobilize nutrients such as Pi for translocation to sink tissues. Some transcription factors demonstrate transient changes in expression levels, highlighting the complexity of how the senescence program is modulated [20,21].

During the early stage of senescence, when the majority of the leaf is still green, increased expression of transcription factors belonging to the *NAC* family occurs [21]. The *Arabidopsis* NAC transcription factor *AtNAP (NAC2)* has been implicated in regulating the onset of senescence [24], as an *AtNAP* null mutant presented a delay in leaf senescence as well as a strong reduction in the expression of the senescence-specific marker gene *SAG12* [29]. NAC transcription factors are also active in abscisic acid (ABA)-inducible gene expression, which may account for the increase in expression observed for ABA-related genes during the onset of senescence [20,21]. Genes related to jasmonic acid (JA) signaling, including those involved in producing JA as well as potential targets of this hormone, are also up-regulated [21]. The increased expression of the NAC transcription factor family, and the resulting influence on hormone metabolism, could have many potential downstream targets. One such target is *ORE1*, the expression of which increases during early senescence [20]. In turn, *AtORE1* promotes the expression of *AtBFN1*, a nuclease that breaks down single-stranded nucleic acids [30], thus contributing to the remobilization of Pi from macromolecules. In addition, *AtORE1* also promotes the expression of *AtSWEET15/SAG29*, a gene involved with sucrose transport, and *AtSINA1*, a gene involved in protein ubiquitination [30], implicating *AtORE1* in the regulatory network of remobilizing nutrients other than Pi as well.

As leaf senescence progresses, the transcriptome profile changes. Many of the same transcription factor families are represented, such as the *NAC* family, while other transcription factors show an enhanced representation, such as the *WRKY* transcription factor family [21]. *WRKY53* is induced by senescence [31] and inhibiting its function delays leaf senescence [32]. *WRKY53* is activated by an upstream mitogen-activated protein kinase kinase kinase (MEKK1), which can bind to the *WRKY53* promoter as well as phosphorylate the WRKY53 protein to regulate the DNA-binding activity of this transcription factor. [33]. Downstream of *WRKY53* are more than 60 putative target genes, including other members of the *WRKY* gene family [32], which may suggest that WRKY53 acts as a regulator of a transcription factor signaling cascade. Another potential target of *WRKY53* is *SQD1* [32], a gene involved in sulfolipid biosynthesis. As Pi is being recycled from phospholipid membranes, the phospholipids can be replaced by sulfolipids to maintain membrane integrity [34]. *WRKY6* is also strongly induced in senescing leaves [35]. *WRKY6* can both activate and repress other *WRKY* genes, and also directly activates the *SIRK/FRK1* promoter, involving *WRKY6* in the signaling cascades that modulate the senescence program [35]. Interestingly, *WRKY6* is also involved in the regulation of *PHO1* expression during nutritional Pi deprivation [36]. PHO1 participates in the Pi translocation process, either as a transporter or as a regulator of Pi transporters belonging to the *Pht1* gene family in *Arabidopsis* [36]. One may thus hypothesize that *WRKY6*, and in turn, *PHO1*, are involved in Pi transport during leaf senescence as well. During mid-senescence, an increase in the expression of genes involved in membrane lipid degradation, cell wall degradation, and metal ion binding is observed [21,22], indicating the continued catabolism of macromolecules to free nutrients such as Pi for transport to other tissues.

The transition into late senescence, when the majority of the leaf becomes yellow, also exhibits a unique pattern of gene expression as compared to early or mid-senescence. In terms of hormones, ABA and ethylene levels increase within the leaf [21]. Both of these hormones positively regulate the *AtORE1* gene as well as the *BFN1* nuclease [30], which may indicate that nuclease activity is augmented during the final stage of senescence to help scavenge Pi from any remaining DNA or RNA. Genes related to metal ion binding and nutrient transport remain up-regulated, presumably to maximize the redistribution of nutrients from source to sink tissues before the integrity of the cell is lost and tissue death occurs [21,22].

**Figure 1:**
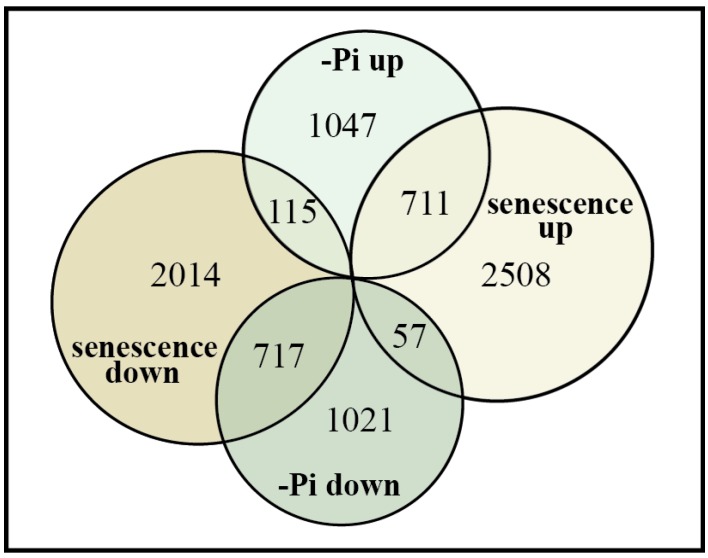
Transcript profiles of Pi-deficient shoots and senescing leaves share overlapping gene expression changes in the model plant *Arabidopsis thaliana*. Numbers of down-regulated or up-regulated genes during leaf senescence [21] or during Pi-deprivation [37] are indicated. Figure modified from [5].

The genome-wide transcriptomic studies have revealed the sheer complexity and scale of differential gene expression involved in the senescence program (Figure 1). Although these studies have outlined changes in gene expression across many different elements of potential signal cascades, more research is required to tease apart the details of the connections and consequences of these expression changes, particularly because changes in gene activity and transcript abundance do not always directly correlate with changes in accumulation or activity of the encoded protein. A key challenge for future research includes large-scale proteomic studies to determine senescence-specific changes at the protein level, including crucial post-translational protein modifications such as phosphorylation or ubiquitination. With the potential targets of senescence control identified by these large-scale studies, a more directed approach toward these targets can provide a clearer picture to piece together the intricate processes that initiate and modulate the leaf senescence program.

### 2.2. Transcriptome Changes during Leaf Senescence versus Nutritional Pi-Deficiency

A key challenge in the quest to understanding plant metabolism and stress response involves teasing apart the mechanism by which plants can respond, both transcriptionally and metabolically, to a diverse array of environmental stimuli and stressors. Of particular interest are similarities or differences that exist in the control of the senescence program *versus* the Pi-starvation response, as both of these processes share common goals regarding cellular Pi metabolism, which is to liberate and mobilize Pi to tissues where it is needed. Under both circumstances, the genes involved include those encoding ribonucleases (RNases), lipid remodeling enzymes, purple acid phosphatases (PAPs), and Pi transporters (Table 2). However, obvious differences between the two processes also exist. While leaf senescence is an organ-specific event, taking place in only specific leaves, the Pi-starvation response involves the entire plant, with tissue-specific gene expression and metabolic changes taking place in both the roots and shoots [1,38]. To determine the extent of transcriptome overlap between Pi-deficiency and senescence, transcript profiles of senescing *Arabidopsis* leaves and Pi-starved shoots were recently compared [5]. A considerable overlap in differentially expressed genes during Pi-starvation and senescence was observed, such that 711 (or 38%) of the 1873 genes up-regulated in shoots during Pi-deficiency were also up-regulated during leaf senescence, whereas 717 (or 40%) of the 1795 genes that were down-regulated during Pi-deficiency were also down-regulated during leaf senescence (Figure 1).

At the heart of the *Arabidopsis* Pi-starvation response is the transcription factor PHR1, a member of the MYB-CC transcription factor family [37,39,40]. *PHR1* is not transcriptionally responsive to Pi stress, but is instead thought to be activated by sumoylation, upon which it acts as a master controller of the Pi-starvation response by transcriptionally activating a complement of genes, evidenced by many genes belonging to the Pi-starvation response containing a PHR1 binding site [37]. These target genes include other transcription factors, such as those belonging to the *WRKY* family; Pi transporter genes, such as those belonging to the *Pht1* family; lipid remodeling genes, including those encoding phospholipases; genes involved in sulfolipid biosynthesis; and genes encoding specific RNase and PAP isozymes [37,39]. Furthermore, mutant plants lacking a functional *PHR1* gene do not have the ability to mount the proper Pi-starvation response, corroborating the importance of this transcription factor as the regulator for adaptability to Pi-deficient conditions [37]. Thus, it will be of great interest to determine whether PHR1 is also involved in the control of Pi remobilization during leaf senescence.

Indeed, many *Arabidopsis* genes induced during Pi-starvation or leaf senescence contain the PHR1 binding site; these include *RNS1*, *RNS2*, *Pht1;5*, *SQDI*, and *PAP17* [39]. However, differences in transcriptional control of these two processes are also evident. For example, AtPAP26 is an *Arabidopsis* PAP isozyme that plays an essential role in Pi scavenging during leaf senescence and Pi-deprivation [7,41,42,43,44,45]. Interestingly, AtPAP26 appears to be differentially regulated in each of the respective scenarios. During leaf senescence, the *AtPAP26* gene is up-regulated, with an increase in the number of *AtPAP26* transcripts accumulating as compared to non-senescing leaves [7]. Conversely, *AtPAP26* transcript levels appear to remain relatively unchanged during the Pi-starvation response [43], with the marked up-regulation in AtPAP26 abundance and activity being instead attributed to post-transcriptional control mechanisms, such as mRNA translation and protein turnover. *AtPAP26* highlights how differential regulation can exist for genes common to both the Pi-starvation response and leaf senescence. A challenging goal will be to determine how plants control these two similar processes, and where these control elements overlap or differ.

**Table 2:** Genes involved in Pi scavenging during nutritional Pi deprivation and/or leaf senescence.

Phosphate Source	Gene	Species (Common Name)	Proposed Function (s)	Reference
Nucleic acids	*RNS2*	*Arabidopsis thaliana* (thale cress)	class II RNase Housekeeping rRNA degradation	[46,47,48]
	*NGR2*	*Nicotiana glutinosa* (tobacco)	class II RNase Housekeeping rRNA degradation	[49]
	*AhSL28*	*Antirrhinum* (snapdragon)	class II RNase Housekeeping rRNA degradation RNA degradation during Pi starvation RNA degradation during senescence response	[50]
	*RNaseLER*	*Solanum lycopersicum* (tomato)	class II RNase Housekeeping rRNA degradation	[51,52]
	*RNaseLX*	*Solanum lycopersicum* (tomato)	intracellular class I RNase RNA degradation during xylem differentiation RNA degradation during leaf abscission	[53,54,55,56]
	*RNaseLE*	*Solanum lycopersicum* (tomato)	extracellular class I RNase RNA degradation during sieve element development RNA degradation during mechanical wounding response	[53,54,57]
	*ZRNase I*	*Zinnia elegans* (common zinnia)	extracellular class I RNase RNA degradation during tracheary element differentiation	[58]
	*RNS1*	*Arabidopsis thaliana* (thale cress)	extracellular class I RNase RNA degradation during Pi-starvation RNA degradation during senescence	[7,41,47]
	*NvRN1*	*Nepenthes ventricosa* (tropical pitcher plant)	extracellular class I RNase RNA degradation from insect prey	[59]
	*BFN1*	*Arabidopsis thaliana* (thale cress)	type I nuclease Nucleic acid degradation during PCD	[60,61,62]
	*LeNUC1*	*Solanum lycopersicum* (tomato)	type I nuclease Nucleic acid degradation during senescence	[63]
Phospholipids	*PLA_1_ gene AF026480*	*Dianthus caryophyllus* (carnation)	Phospholipase A_1_ Hydrolysis of phospholipids Promote senescence progress	[64,65]
	*NPC4*	*Arabidopsis thaliana* (thale cress)	Phospholipase C Phospholipid hydrolysis during Pi-starvation	[66]
	*PLDα*	*Arabidopsis thaliana* (thale cress)	Phospholipase Hydrolysis of phospholipids Promote senescence progress	[67]
	*SQD1*	*Arabidopsis thaliana* (thale cress)	Sulfoquinovosyldiacylglycerol Sulfolipid biosynthesis	[32]
Other Pi-monoesters	*AtPAP26*	*Arabidopsis thaliana* (thale cress)	Purple acid phosphatase (dual-targeted to cell vacuole and cell wall/secretome) Scavenge Pi during Pi-starvation Scavenge Pi during senescence	[7,41,42,43,44,45]
	*HpPAP1*	*Hakea prostrata* (harsh hakea)	Purple acid phosphatase Scavenge Pi during senescence	[41]
	*AtPAP17*	*Arabidopsis thaliana* (thale cress)	Purple acid phosphatase Scavenge Pi during Pi-starvation ROS metabolism	[7,41,43,68]
	*AtPPsPase1*	*Arabidopsis thaliana* (thale cress)	HAD pyrophosphatase Scavenge Pi during Pi-starvation	[69]
	*AtPECP1*	*Arabidopsis thaliana* (thale cress)	HAD phosphoethanolamine/phosphocholine phosphatase Scavenge Pi during Pi-starvation Phospholipid degradation	[70]
	*AtSgpp*	*Arabidopsis thaliana* (thale cress)	HAD phosphosugar phosphatase Scavenge Pi during Pi-starvation	[71]
	*LePS2;1*	*Solanum lycopersicum* (tomato)	HAD protein phosphatase Pi signaling during Pi-starvation	[72,73]
	*PvPS2:1*	*Phaseolus vulgaris* (common bean)	HAD protein phosphatase Pi signaling during Pi-starvation	[74,75]

## 3. Catabolism of Macromolecules Frees Phosphate for Remobilization

The downstream targets of the signal cascades initiated by hormones and transcription factors include many hydrolytic enzymes (Table 2) that are responsible for the catabolism of varying cellular components, leading to the death of the cell [21,22]. As this cellular break-down occurs, nutrients, including Pi, are liberated from their respective biomolecules; these nutrients can then be mobilized for transport to sink tissues, particularly developing seeds, where they are re-utilized in synthesizing new macromolecules or various low molecular weight metabolites [4].

### 3.1. Catabolism of Nucleic Acids

Nearly half of the total P present within a healthy leaf exists within nucleic acids; of that, approximately 80% is represented by ribosomal RNA (rRNA) [4]. rRNA levels increase in developing leaves as their capacity for protein synthesis grows, whereas mature leaves experience reduced protein synthesis and thus declining rRNA levels [76,77]. Early leaf senescence brings about the enhanced degradation of rRNA, as well as other RNA molecules, which continues and also expands to include the degradation of DNA in the late stages of senescence [21].

**Figure 2:**
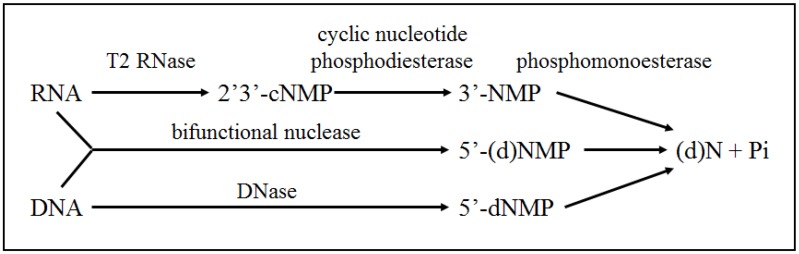
Model of nucleic acid degradation and Pi recycling by nucleolytic enzymes (modified from [79]).

Recycling Pi from the large pool of rRNA is initiated by RNases, such as those from the RNase T2 family. Members of the T2 family are present within the genome of virtually all organisms, demonstrating their essential role in rRNA decay within the cell [46]. The RNase T2 family is composed of two subfamilies: the S-RNases and the S-like RNases. S-RNases are involved in the rejection of self-pollen to establish self-incompatibility for plants belonging to the *Solanaceae*, *Scrophulariaceae*, and *Rosaceae* families [78]. The S-like RNases do not play a role in gametophytic self-incompatibility, but are instead involved with defense and Pi recycling [51]. Transcriptomic studies reveal that S-like RNases are transcriptionally induced during Pi-starvation or senescence. The tomato (*Solanum lycopersicum*) RNases *RNase LE* and *RNase LX*, as well as the *Arabidopsis* RNases *RNS1* and *RNS2*, are transcriptionally induced during Pi-starvation [47,48,53]. In addition, the expression of *RNase LX* and *RNS2* is increased during senescence [48,54]. RNases belonging to the T2 family catabolize RNA into nucleotide monophosphates (NMPs) by way of a 2′,3′-cyclic nucleotide monophosphate intermediate (cNMP) [46]. This cNMP intermediate is then further catabolized by a cyclic nucleotide phosphodiesterase, resulting in an NMP that can act as a substrate for phosphomonoesterases, particularly PAPs, which will liberate Pi for transport to sink tissues [79] (Figure 2).

The S-like RNases can be further categorized as belonging to class I or class II, with class II RNases sharing conserved ancestral characteristics, an intracellular localization, and constitutive expression, suggesting a housekeeping-related role for these enzymes [47,51,53]. Class II RNases, including *RNS2*, appear to be abundantly expressed in most tissues during all stages of plant development, indicating that this class of RNases is essential to the normal growth of the plant. Loss of *RNS2* function in knockout mutants results in a longer half-life for rRNA and the subsequent constitutive autophagy of the tissue under standard growth conditions, further corroborating the importance of class II RNases [46]. Much like the *Arabidopsis* *RNS2*, the tobacco (*Nicotiana glutinosa*) *NGR2* and *Antirrhinum* *AhSL28* also show constitutive expression in diverse tissues [49,50], and *AhSL28* expression displays a distinct increase during Pi-deprivation as well as during leaf senescence [50]. This may indicate that the main function of class II RNases is natural RNA decay during development, but this function can expand to aid in alleviating nutritional stress, such as when Pi is limiting. However, not all class II RNases follow this pattern. The tomato *RNaseLER* is a class II RNase that is expressed in stems, flowers, roots, and leaves [51], but shows the greatest accumulation of expression in the guard cells and trichomes [52]. This RNase does not display a transcriptional response to Pi-starvation, osmotic stress, or drought; in addition, neither ABA nor ethylene treatments stimulated a significant increase in *RNaseLER* expression, which could indicate that this RNase would not be responsive to senescence [52]. Thus, despite the apparent strong genetic conservation among the class II RNases, not all isozymes belonging to this group share a common function in mobilizing Pi during senescence or Pi-deprivation.

In contrast to the class II RNases, the class I RNases display a great diversity, are often secreted into extracellular locations, and are generally expressed sparingly under normal growth conditions, but can be highly induced by various environmental cues such as nutrient deficiency, senescence, osmotic stress, drought, and mechanical wounding [51]. Several class I RNases are transcriptionally induced during different developmental stages in addition to their responsiveness to environmental stressors. Tomato *RNase LX* is sensitive to ethylene treatment, but is also highly induced during xylem differentiation [54,55]; RNase LX has also been implicated in the leaf abscission process, as mutant plants lacking this enzyme where phenotypically characterized by delayed leaf abscission [55]. In contrast, tomato *RNase LE*, is expressed in response to mechanical wounding and ABA treatment, but is localized in the phloem tissue, which may indicate a role in the development of sieve elements [57]. Furthermore, a class I RNase from common zinnia (*Zinnia elegans*), ZRNase I, participates in tracheary element differentiation [58]. Taken together, one may conclude that class I RNases are involved in events of developmental programmed cell death (PCD); considering that leaf senescence is thought to end in PCD, it is reasonable to hypothesize that class I RNases may contribute to PCD during leaf senescence. In fact, several class I RNases show increased mRNA and protein expressions during the course of leaf senescence, such as *RNase LX* and *RNase LE* from tomato [54], as well as *RNS1* from *Arabidopsis* [7,41,47]. Class I RNases are directly involved in scavenging nutrients, such as Pi, from the environment, as exemplified by carnivorous plants. *Drosera adelae*, a trap leaf plant, secretes a sticky substance containing S-like RNases to scavenge Pi from the insect prey it has trapped in its leaves [80]. Similarly, NvRN1, an S-like RNase from the pitcher plant *Nepenthes ventricosa*, was found in the pitcher fluid of this plant, likely to free nutrients from the RNA of its unlucky prey [59]. Expression of *RNase LX*, *RNase LE*, and *RNS1* also increases during periods of Pi-deprivation, further indicating that RNase activity can be essential for scavenging Pi from macromolecules, in this case to aid in the mitigation of the nutrient deficiency [47,48,53]. Although non-carnivorous plants may not secrete RNases onto the surface of their leaves or into a pitcher fluid, many class I RNases are targeted for secretion to extracellular locations such as the apoplast and rhizosphere [42,51]. In *Arabidopsis*, RNases are secreted by the roots to free Pi from nucleic acids present in the soil, demonstrated by the ability of the plant to grow on exogenous nucleic acids as its sole source of P nutrition [42]. In the apoplast, secreted RNases are also believed to scavenge Pi from intracellular oligonucleotides that have leaked through the plasma membrane [7,41]. This function may be particularly relevant during leaf senescence, as the catabolism of membrane lipids may facilitate the movement of small oligonucleotides and Pi-esters into the apoplast [81]. However, not all class I RNases are extracellularly targeted. In contrast to RNase LE and RNS1, which are both secreted, RNase LX is restricted to the endoplasmic reticulum [53,56], demonstrating that the intracellular pools of RNA are also subject to recycling during senescence or Pi-deprivation.

As senescence advances and cell death is imminent, the degradation of nucleotides expands from the RNA pool to include DNA catabolism [21], which is carried out by nucleases. The *Arabidopsis* *Bifunctional Nuclease I* (*BFN1*) gene encodes a type 1 nuclease, and is induced during the senescence of leaves, flowers, and stems, as well as during other PCD events such as xylem development in young seedlings [60,61,62]. As a bifunctional nuclease, BFN1 can degrade both single stranded RNA and DNA (Figure 2), and co-localizes with fragmented nuclei in senescing tobacco protoplasts. This observation suggests that AtBFN1 is actively involved in DNA catabolism during senescence [60], although the expression pattern of the *BFN1* promoter implicates this gene’s involvement in other PCD-associated developmental processes as well [82]. As mentioned previously, *BFN1* gene activity is positively regulated by the senescence-associated NAC transcription factor, *AtORE1*, as well as by ethylene treatment, further promoting the role of *BFN1* in senescence and PCD events [30]. Much like *BFN1* in *Arabidopsis*, a tomato nuclease, *LeNUC1*, is transcriptionally induced during leaf senescence and has a protein product that exhibits both RNase and DNase activity [63]. Interestingly, *LeNUC1* expression in young leaves is induced upon exposure to ethylene, a senescence-promoting hormone [63]. The similarities between the *Arabidopsis* and tomato nucleases reinforce the notion that nuclease activity is integral to the leaf senescence program, such that it is conserved across species.

### 3.2. Catabolism of Lipids

**Figure 3:**
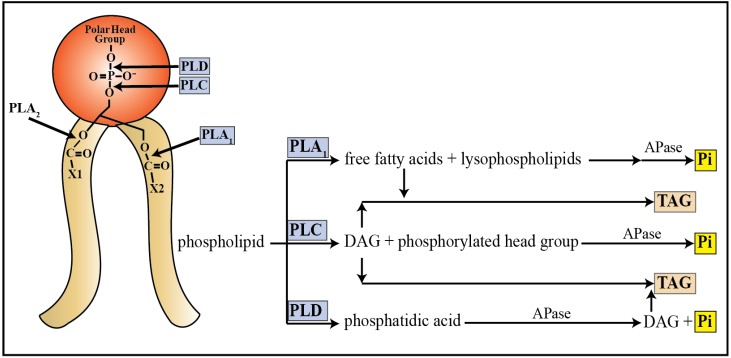
Model of glycerophospholipid degradation and Pi recycling by phospholipase enzymes. Enzymes belonging to the phospholipase A_1_ (PLA_1_), phospholipase C (PLC), and phospholipase D (PLD) groups have been implicated in recycling Pi during times of nutritional Pi-starvation or leaf senescence. DAG, diacylglyceride; TAG, triacylglyceride; X1 and X2, long chain fatty acids.

A second pool of P within a senescing leaf is amphipathic membrane glycerophospholipids, consisting of a pair of hydrophobic long-chain fatty acids esterified to the C-1 and C-2 positions of glycerol, together with a polar head group attached via a phosphodiester linkage to glycerol’s C-3 position. During normal plant growth, a constitutive turnover of membrane lipids occurs, with approximately 2% of these lipids being replaced each day [83]. However, during senescence, an extensive remodeling of membrane lipids takes place; the rate of fatty acid synthesis declines, while the rate of membrane lipid catabolism rises, ultimately resulting in a net loss of membrane lipids and disruption of membrane integrity [83,84]. This membrane remodeling is so extensive that an 80% decrease in total fatty acid content was observed during the natural senescence of *Arabidopsis*, Brachypodium (*Brachypodium distachyon*), and switchgrass (*Panicum virgatum*) [85]. There are four main groups of plant phospholipases that work to catabolize glycerophospholipids (Figure 3); of these four groups, three have been implicated in the senescence program or Pi-starvation response [86]. Each class of phospholipase shows specificity regarding the site of cleavage on the phospholipid molecule, resulting in a variety of hydrolytic products [86]. Further catabolism of these hydrolytic products can release Pi from the polar head group for transport to sink tissue (Figure 3).

Lipases belonging to the phospholipase A_1_ (PLA_1_) family cleave phospholipids to produce lysophospholipids and free fatty acids. In carnation (*Dianthus caryophyllus*), an increase in transcriptional expression of a *PLA_1_* gene (AF026480) occurs just as the flowers begin to senesce, as well as after exposure to ethylene [64]. Expression of the orthologous *PLA_1_* gene in *Arabidopsis* showed a similar pattern, and was in stark contrast to a different *PLA_1_* gene involved in the turnover of lipids during the growth phase, which demonstrated a marked decline in expression as senescence was initiated [65]. Thus, senescence-inducible PLA_1_ isozymes could play a role in mediating the onset of senescence, which is further corroborated by the delayed senescence phenotype observed in transgenic *Arabidopsis* that had a reduced expression of this PLA_1_ isozyme [86]. It was postulated that the α-linoleic acid released during membrane catabolism could promote the synthesis of JA, a plant hormone implicated in the initiation of the senescence program [22]. One of the earliest signs of senescence is leaf yellowing, which indicates a dismantling of chlorophyll and the photosynthetic machinery; thylakoid membranes, being a key component of the photosynthetic apparatus, may be an early target of the senescence program. As such, this PLA_1_ isozyme may initiate thylakoid membrane catabolism at the onset of senescence.

Members of the phospholipase C (PLC) family produce diacylglycerol (DAG) and a phosphorylated head group as hydrolytic products of glycerophospholipid metabolism, whereas members of the phospholipase D (PLD) family produce phosphatidic acid (Figure 3) [86]. Both of these phospholipase families are involved in stress responses, including nutrient starvation and leaf senescence. In *Arabidopsis*, the PLC isozyme *NPC4* is up-regulated during Pi-starvation, and disrupting NPC4 function results in a striking reduction of PLC activity in response to Pi-deficiency [66]. Therefore, NPC4 may aid in scavenging Pi to relieve the nutritional stress. It would be of interest to determine whether this function is carried over under conditions of leaf senescence. Likewise, members of the PLD family have been implicated in glycerophospholipid catabolism during Pi-starvation, but also play a direct role in the leaf senescence program [86]. When the expression of the *Arabidopsis* PLD isozyme *PLDα* was suppressed, the rate of senescence in ABA- and ethylene-treated leaves was delayed [67,87]. As such, PLDα may participate in liberating Pi from glycerophospholipids, as well as modulate the progression of senescence, much like the PLA_1_ family.

The DAG released from the hydrolytic action of PLC enzymes, as well as from the further catabolism of phosphatidic acid produced by PLD enzymes (Figure 3), can then be converted into triacylglycerol (TAG). In *Arabidopsis*, TAG accumulation occurs with advancing leaf senescence [83,88]. This observation coincides with the increased pattern of diacylglycerol acyltransferase (*DGAT10*) expression, which is involved in TAG biosynthesis [80]. As the catabolism of thylakoid membranes occurs during early senescence, TAG is synthesized in the chloroplasts to sequester the released DAG and fatty acids [88]. The production of TAG may act as a buffer during the earliest stage of senescence to prevent the premature degradation of cell membranes, as free fatty acids act as a substrate for membrane lipoxygenases whose lipid peroxidation action is thought to contribute to the deterioration of the membrane [84]. As such, TAG accumulation during the dismantling of the photosynthetic machinery could contribute to the prolonging of membrane integrity, therefore allowing more time for nutrients such as Pi to be recycled out of the senescing leaf before cell death occurs.

In the later stages of senescence, lipid remodeling moves beyond the thylakoid membranes to target other cellular membranes as well. During this time, genes encoding enzymes required for β-oxidation and the glyoxylate pathway are up-regulated, reflecting the process of metabolizing freed fatty acids, as well as the sequestered TAG, into carbohydrate molecules such as sucrose for transport to sink tissues [22]. Also up-regulated during this time may be genes involved in sulfolipid biosynthesis, such as *SQD1* [32]. This gene was identified as a potential target of the transcription factor *WRKY53*, the expression of which is up-regulated during early to mid-senescence [22,32]. Similarly, the replacement of glyerophospholipids with sulfolipids occurs during Pi-deficiency to liberate Pi from membrane lipids and replace them with lipids containing a different polar head group, such as sulfate [34]. The targeting of sulfolipid biosynthetic genes during senescence likely reflects the active recycling of Pi from glycerophospholipids, as well as the importance of maintaining relative membrane integrity throughout the senescence process so as to facilitate nutrient recycling.

### 3.3. Other Phosphate Pools

A final cellular pool of P includes Pi-monoesters and phosphoanhydrides, such as phosphorylated proteins, phosphorylated sugars, pyrophosphate, and nucleoside-, di-, and tri-phosphates [4]. These P-containing compounds are the target of phosphatases, which liberate a Pi group from the ester. It has been widely established that vacuolar and secreted PAPs play an important role in scavenging and remobilizing Pi during nutritional Pi-deficiency [1], but the action of PAPs has only been recently linked to Pi metabolism and recycling during leaf senescence as well [7,41].

Members of the plant PAP family are generally effective at hydrolyzing Pi from a broad range of Pi-monoesters and anhydrides and exhibit optimal activity at an acidic pH [1]. Plant PAP families are relatively large, with the *Arabidopsis* genome encoding 29 putative *PAP* genes, each responsive to a variety of developmental and environmental cues [89]. Consequently, PAPs may have diverse roles during development and stress responses, such as during leaf senescence. AtPAP26 is a well-characterized *Arabidopsis* PAP isozyme shown to have a key role both during Pi-deprivation and leaf senescence. This enzyme displays high catalytic efficiency with numerous Pi-ester substrates over a broad pH range [43], allowing it to function effectively both inside the cell as well as in the extracellular environment as AtPAP26 is dual-targeted to both the vacuole and cell wall/apoplast [42,44,45]. AtPAP26 is crucial to the successful acclimation of *Arabidopsis* to Pi-deficient conditions; mutant plants lacking this enzyme have greatly reduced acid phosphatase (APase) activity, as well as impaired growth in Pi-deficient media [42,44]. APase activity shows a marked increase in both intracellular and cell wall extracts of senescing leaves as compared to non-senescing leaves of wild type *Arabidopsis*. However, in an *atpap26* knockout line, a very small increase in senescence-inducible APase activity was observed [7,41]. The loss of AtPAP26 also resulted in delayed leaf senescence and severely reduced P resorption efficiency, from 70% of total P remobilized in the wild type control to 15% remobilized from the senescing leaves of the *atpap26* mutants [7]. Just as AtPAP26 is up-regulated and dual-targeted during Pi deprivation, the protein abundance and activity of AtPAP26 is also markedly enhanced in both the vacuole and cell wall of senescing *Arabidopsis* leaves [7,41]. It was hypothesized that secreted AtPAP26 of senescing leaves scavenges any Pi-esters that may have escaped past the leaky plasma membrane in order to maximize Pi remobilization. Furthermore, a functional ortholog of AtPAP26 appears to be responsible for the observed increases in intracellular and cell wall APase activity of senescing leaves of harsh hakea (*Hakea prostrata*), an extremophile species of the nutrient-impoverished soils of Western Australia and whose leaves display remarkably efficient Pi remobilization capabilities (Table 1) [11,41]. This observation provides additional evidence that PAP26 orthologs play a predominant Pi recycling role during leaf senescence.

Another *Arabidopsis* PAP isozyme implicated in leaf senescence is AtPAP17 [7,41]. AtPAP17 was first characterized as a protein responsive to Pi-deprivation, as its activity at both the transcript and protein level is increased under this condition [43,68]. *AtPAP17* expression is also induced by stimuli such as salinity and hydrogen peroxide, which may relate more to the alkaline peroxidase activity that this enzyme also possesses, as this activity could contribute to the metabolism of reactive oxygen species [68]. *AtPAP17* transcripts increased nearly 24-fold in senescing leaves as compared to non-senescing leaves; combined with the responsiveness of *AtPAP17* to ABA, a hormone thought to promote senescence, this observation implies a potential role for this PAP isozyme in the senescence program [7,68]. However, more research must be done to ascertain whether AtPAP17’s marked transcriptional induction during leaf senescence is paralleled by enhanced AtPAP17 protein accumulation and enzymatic activity. Transcriptomic studies have also revealed that *AtPAP12*, a major PAP isozyme that is secreted with AtPAP26 to scavenge Pi in the apoplast during *Arabidopsis* Pi deprivation [42], is transcriptionally induced during leaf senescence [90]. However, AtPAP12 does not appear to be a major player in Pi remobilization either within the cell or cell wall during leaf senescence [7,41], further highlighting how transcriptional induction is not always actualized at the protein level.

A distinct class of phosphatases separate from the PAPs that also show sensitivity to Pi-status are those belonging to the haloacid dehalogenase (HAD) superfamily, which includes a diverse assortment of enzymes thought to be present in all eukaryotes [91]. The majority of the enzymes in this superfamily are involved in phosphoryl transfer reactions and include phosphatases, ATPases, dehalogenases, and sugar phosphomutases. Several members of the HAD family are transcriptionally induced in response to Pi-deprivation. In *Arabidopsis*, the *AtPPsPase1* gene is highly activated by Pi-starvation and encodes a pyrophosphatase, suggesting that the function of this gene is to liberate Pi from pyrophosphate when free Pi levels within the cell become too low [69]. AtPECP1 is a Pi status-sensitive phosphoethanolamine/phosphocholine phosphatase with a broad pH optimum that participates in phospholipid degradation [70]. Similarly, AtSgpp acts to liberate Pi from phosphosugars, such as glucose-6-phosphate [71]. Thus, members of the HAD superfamily act to mobilize Pi from a wide range of Pi-esters so as to alleviate nutritional stress. In addition, transcriptomic studies have revealed that many genes belonging to the HAD superfamily are up-regulated during senescence [21,92], which opens the door to a potential role for these hydrolases in Pi liberation and remobilization in the aging leaf. However, further research is required to confirm such a role.

Interestingly, several HAD members also show a potential signaling capacity in response to changes in Pi status. *LePS2;1* is a protein phosphatase gene from tomato that is induced during Pi-starvation, but encodes a protein that has very low phosphatase activity against *para*-nitrophenyl phosphate, a common synthetic substrate for phosphatases [72]. However, *LePS2;1* effectively dephosphorylates a synthetic serine/threonine peptide; additionally, the overexpression of *LePS2;1* in tomato plants resulted in an increase in anthocyanin accumulation as well as APase activity under Pi-sufficient conditions [73]. Similarly, the expression of *PvHAD1*, a serine/threonine phosphatase gene from common bean (*Phaseolus vulgaris* L.) was observed to be highly sensitive to Pi supply, but its protein product showed no phosphatase activity with *p*-nitrophenyl phosphate as a substrate [74]. Another HAD ortholog from bean, *PvPS2:1*, was overexpressed in *Arabidopsis*. The resulting transgenic line showed enhanced expression of two Pi starvation responsive genes as well as augmented phosphatase activity [75]. These representative results from tomato and bean suggest that some HAD members may not play a direct role in liberating Pi during nutritional stress, but may instead be part of the signaling process, activating the genes and proteins that participate in a more direct Pi-scavenging role. Although leaf senescence may not reflect the same conditions as Pi-starvation, it could still implicate changes in Pi status as macromolecules are broken down and nutrients are moved out of the cell. One could speculate that HAD genes may be involved in signaling during senescence as well, although more research is required to explore this capability.

## 4. Phosphate Transport from Senescing Leaves to Growing Tissue

**Figure 4:**
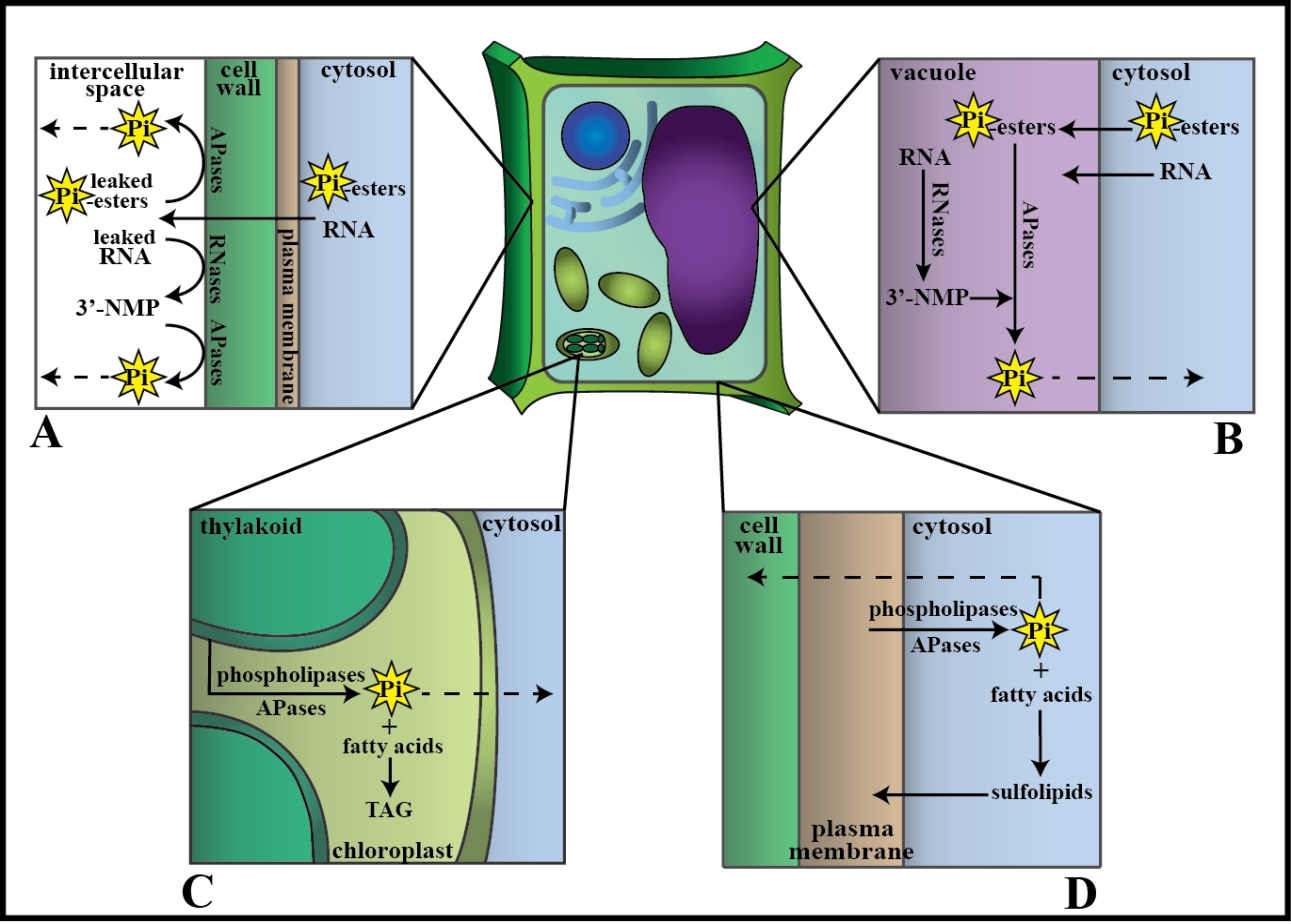
Simplified model of Pi remobilization in a senescing plant cell. (**A**) RNA oligonucleotides and Pi-esters leak through the partially degraded plasma membrane beyond the cell wall, where they are respectively hydrolyzed by cell wall-localized RNases and acid phosphatases (APases). Dotted lines denote the transport of Pi out of the senescing cell to other tissues. (**B**) Intracellular RNases and APases hydrolyze RNA and Pi-esters, respectively, in the vacuole. (**C**) Phospholipids are metabolized by the action of phospholipases and APases in the chloroplasts; Pi is released and free fatty acids are sequestered as triacylglycerol (TAG). (**D**) Phospholipases and APases liberate Pi from phospholipids of the plasma membrane, with some of the resulting free fatty acids being incorporated back into the membrane as sulfolipids.

Upon the catabolism of the various P-containing biomolecule pools within a senescing leaf, as summarized in Figure 4, the released Pi must be exported for transport to sink tissues. Pi transporters take on this role, not only to aid Pi remobilization from older tissues, but also to recover any Pi in the vascular tissue that could be lost via guttation or leakage across the plasma membrane to the apoplast, and to unload the Pi into symplastically isolated tissues [93].

A well-studied group of Pi-transporters belong to the *Pht1* family, a collection of Pi:H^+^ symporters, the majority of which have a high affinity for Pi and are expressed in roots [94]. These transporters are often induced during nutritional Pi-deficiency, when the plant must assimilate Pi against a very steep concentration gradient from the soil into the roots [1,94]. Despite the vast majority of Pi transporters being expressed in the roots, as their central role is Pi uptake from the soil, many transporters are expressed in shoots as well, implicating a Pi distribution function [94]. Low affinity transporters such as *AtPht1;5* from *Arabidopsis* [95], *HORvu;Pht1;6* from barley (*Hordeum vulgare* L.) [96], and *OsPht1;1* and *OsPht1;2* from rice [97,98], are predominantly expressed in the aerial tissues of the plant. These transporters potentially serve the purpose of moving Pi against a less steep gradient, such as from senescing leaves into the phloem, as evidenced by *HORvu;Pht1;6* expression being strongest in old leaves [96] and *AtPht1;5* overexpression resulting in enhanced Pi accumulation in siliques [95]. In addition to being expressed in the roots, some high affinity Pi transporters, including rice *OsPht1;8*, are expressed in shoot tissues, and therefore may also contribute to Pi distribution within the plant; when *OsPht1;8* was functionally knocked down, the translocation of Pi from the panicle axis to the grain was impaired [99]. This illustrates that some of the transporters previously implicated in Pi acquisition from the soil have the potential to be involved in other aspects of Pi metabolism as well.

In addition to the *Pht1* family of Pi transporters, members of the *Pht2* family and *SPX* superfamily are directly involved with Pi movement as transporters, as well as indirectly by functioning as control elements in the transport mechanism. The *Arabidopsis* *AtPht2;1* encodes a low-affinity Pi transporter and is chiefly expressed in green tissues, co-localizing with chloroplasts [100,101]. Mutant plants not expressing this gene display impaired translocation of Pi within leaves when subjected to Pi-deficient conditions [101]; it is reasonable to postulate that a similar phenotype may arise under conditions of senescence. The *AtPht2;1* ortholog in wheat, *TaPht2;1*, is also strongly expressed in leaves and detected in the chloroplast [102]. A highly P-efficient wheat cultivar exhibited enhanced *TaPht2;1* expression in leaves relative to a less P-efficient cultivar, suggesting that this transporter may contribute to the overall PUE of the plant [103]. AtPHO1 is a SPX protein from *Arabidopsis* thought to be required to load Pi into the xylem. *AtPHO1* expression in leaves is up-regulated following treatment with ABA [104,105], providing evidence for a possible function of this gene during the senescence program. *OsSPX-MFS1* encodes a Pi transporter and is expressed in the leaves of rice. Knocking down the expression of this gene results in impaired Pi remobilization, where the total concentration of P in the older leaves of the knock-down line was approximately two times greater than a wild type control [106]. This indicates that the OsSPX-MFS1 transporter may be directly involved in exporting Pi out of aging leaves. On the regulatory side of transport, AtNLA, a RING-type ubiquitin E3 ligase belonging to the SPX superfamily from *Arabidopsis*, is involved in mediating the action of Pi transporters. AtNLA acts to degrade Pht1 Pi transporters by directing the polyubiquitination of the plasma membrane-localized PHT1 proteins [107]. The regulatory action of AtNLA is crucial to the proper progression of the senescence program, as an *AtNLA* knockout mutant senesced earlier and more rapidly than a wild type control [108], a phenotype that resulted from too many Pi transporters being present, ultimately causing Pi toxicity [109]. Consequently, both Pi transporters and their regulatory controls are needed to achieve efficient Pi remobilization during leaf senescence.

## 5. Concluding Remarks

Leaf senescence is a critical component of the life cycle for a plant as it allows for the investment of resources into new tissues. Central to this is the ability to remobilize essential macronutrients, such as Pi, from the aging leaf to sink tissues. The Pi remobilization process requires the coordinated function of hormones and transcription factors to initiate signaling cascades, resulting in the action of hydrolases that can liberate Pi from P-containing molecules including nucleic acids, phospholipids, phosphoanhydrides, and Pi-ester-containing metabolites. Pi transporters then facilitate Pi translocation from the old leaf to developing tissues such as expanding leaves and developing seeds. Although recent research has explored various components of Pi remobilization and transport, particularly at the transcriptome level, more directed research is required to fully understand the complex control of the leaf senescence program, as well as similarities and differences compared with the Pi-starvation response, both in terms of the control and execution. Such research needs to include large-scale proteomic studies, where senescence-induced changes in protein expression and their post-translational modifications are explored. With a more complete and integrative knowledge of the Pi recycling mechanism, biotechnological advancements regarding crop PUE may be within reach. It would be of great interest to explore how common crop species vary in PUE, and how their PUE may be augmented so as to reduce the considerable cost and pollution associated with the widespread use of non-renewable Pi-containing fertilizers.
